# Identification of Autophagy Related circRNA-miRNA-mRNA-Subtypes Network With Radiotherapy Responses and Tumor Immune Microenvironment in Non-small Cell Lung Cancer

**DOI:** 10.3389/fgene.2021.730003

**Published:** 2021-09-09

**Authors:** Liyuan Fan, Baosheng Li, Zhao Li, Liang Sun

**Affiliations:** ^1^Cheeloo College of Medicine, Shandong University, Jinan, China; ^2^Department of Radiation Oncology, Shandong Cancer Hospital and Institute, Shandong First Medical University and Shandong Academy of Medical Sciences, Jinan, China; ^3^Shandong Yidian Gene Technology Co., Ltd., Jinan, China; ^4^College of Artificial Intelligence and Big Data for Medical Sciences, Shandong First Medical University & Shandong Academy of Medical Sciences, Jinan, China

**Keywords:** non-small cell lung cancer, autophagy, radiotherapy sensitivity, tumor immune microenvironment, competing endogenous RNA

## Abstract

Lung cancer (LC) is one of the most frequently diagnosed cancers and the leading cause of cancer death worldwide, and most LCs are non-small cell lung cancer (NSCLC). Radiotherapy is one of the most effective treatments for patients with lung cancer, either alone or in combination with other treatment methods. However, radiotherapy responses vary considerably among NSCLC patients. The efficacy of radiotherapy is influenced by several factors, among which autophagy is of importance. Autophagy is induced by radiotherapy and also influences cell responses to radiation. We explored the clinical significance of autophagy-related genes (ARGs) and gene sets (ARGSs) and the underlying mechanism in NSCLC patients treated with radiotherapy. First, differentially expressed ARGs (SNCA, SESN3, DAPL1, and ELAPOR1) and miRNAs (miR-205-5p, miR-26a-1-3p, miR-6510-3p, miR-194-3p, miR-215-5p, and miR-375-3p) were identified between radiotherapy-resistant and radiotherapy-sensitive groups. An autophagy-related radiosensitivity risk signature (ARRS) by nine ARmRNAs/miRNAs and an autophagy-related overall survival risk signature (AROS) by three ARmRNAs were then constructed with estimated AUCs of 0.8854 (95% CI: 0.8131–0.9576) and 0.7901 (95% CI: 0.7168–0.8685), respectively. The correlations between ARGSs or prognostic signatures and clinicopathological factors, short-term radiotherapy responses (radiotherapy sensitivity), long-term radiotherapy responses (overall survival), and immune characteristics were analyzed. Both ARGSs and prognostic signatures were related to immune checkpoint inhibitors (ICIs), infiltration of tumor-infiltrating immune cells (TIICs), and the activity of the cancer immune cycle. Finally, after target prediction and correlation analysis, circRNA (hsa_circ_0019709, hsa_circ_0081983, hsa_circ_0112354, hsa_circ_0040569, hsa_circ_0135500, and hsa_circ_0098966)-regulated miRNA/ARmRNA axes (miR-194-3p/SESN3, miR-205-5p/ELAPOR1, and miR-26a-1-3p/SNCA) were considered potential modulatory mechanisms by influencing the regulation of autophagy, macroautophagy, and chaperone-mediated autophagy.

## Introduction

With an estimated 2.2 million new cases and 1.8 million deaths, lung cancer (LC) is one of the most frequently occurring cancer and the leading cause of cancer death according to the most recent global cancer statistics (Sung et al., 2021). In most countries, the 5-year survival rate of patients with LC is only 10 to 20% during 2010 through 2014 (Allemani et al., 2018). To increase the survival rate of patients, improving therapeutic effectiveness is as important as early screening. Radiotherapy is one of the most effective treatments for patients with LC, either alone or in combination with other treatment methods. However, because of individual heterogeneity, radiotherapy responses vary among patients, especially in those with non-small cell lung cancer (NSCLC) (Baker et al., 2016), which accounts for 80% of LC. An important focus of radiation oncology research is to predict radiotherapy responses by using molecular analysis.

Autophagy, a major type of programmed cell death, has been generally regarded as a survival or cytoprotective response under stressful conditions, for example, exposure to radiation and chemicals (Murrow and Debnath, 2013). A growing body of evidence indicates that tumor resistance to anticancer therapies, such as radiotherapy, was often associated with the regulation of autophagy (Sharma et al., 2014; Tam et al., 2017). Although no consensus has been reached about the antitumor or protumor action of autophagy induction, autophagy inhibitors or promoters are potential drug-drug or drug-radiation combinations to promote therapeutic efficacy. Thus, understanding the functional relevance of autophagy within radiotherapy is critical to evade resistance and enhance the effects for NSCLC patients. In addition, few studies have discussed the selective types of autophagy, which are highlighted in our study.

Non-coding RNAs (ncRNAs), accounting for 98% of the human genome, mediate protumorigenic/antitumorigenic responses to different cancer therapies (Zhang X. et al., 2020). MicroRNAs (miRNAs) are a family of small ncRNAs of approximately 22 nucleotides that play an important role in biological pathways by silencing mRNAs and regulating the expression of genes posttranscriptionally (Ambros, 2004). Circular RNAs (circRNAs), another type of ncRNA that can act as gene regulators or even be encoded into proteins, also play vital tumor-regulated roles in numerous cancers (Chen, 2020). Many cases have shown that circRNAs can interact with miRNAs and then form a network to regulate cellular physiological and pathological activities (Hansen et al., 2013). Moreover, due to their relatively stable structure, miRNAs and circRNAs can also be used as biomarkers of cancer therapeutic effects.

In the present study, we made full use of publicly available large-scale cancer omics data, mainly The Cancer Genome Atlas (TCGA), to investigate the clinical significance and underlying mechanisms of autophagy-related genes and gene sets in radiotherapy responses of NSCLC patients. First, patients receiving radiotherapy with complete prognostic information were retrieved, and autophagy-related genes (ARGs) and gene sets (ARGSs) were identified. Then, radiotherapy sensitivity- and overall survival (OS)-related risk signatures were generated following the differential analysis of ARGs and miRNAs. Risk signatures were then subjected to correlation analysis of clinicopathologic factors, predictive value of prognosis, and characteristic analysis of the immune microenvironment. Finally, after targeting prediction and correlation analysis of expression levels, a circRNA-miRNA-ARmRNA-ARGS network was constructed to explain the potential regulatory mechanism.

## Materials and Methods

### Schematic Diagram of the Study Design

**FIGURE 1:**
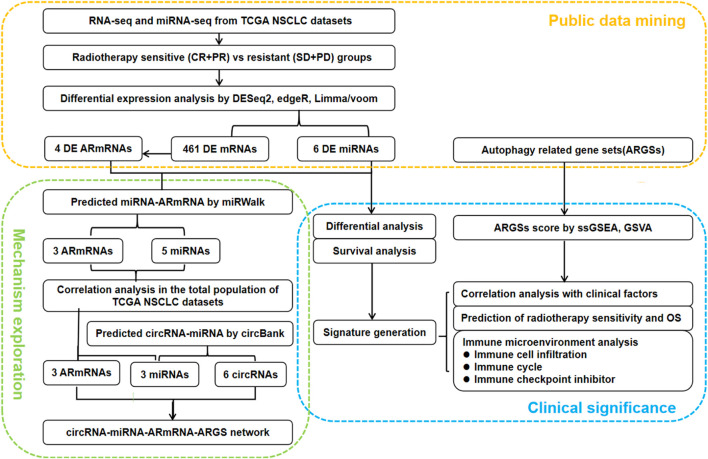
Flowchart of the study design. CR: complete remission/response, PR: partial remission/response, SD: stable disease, PD: progressive disease, DE: differential expressed, ARmRNA: autophagy-related mRNA, NSCLC: non-small cell lung cancer.

As shown in Figure 1, we first mined the public data for our datasets of interest. NSCLC patient information from The Cancer Genome Atlas (TCGA) project was obtained from UCSC Xena^1^. The targeted screening was performed according to the following criteria: (1) patients treated with radiotherapy without additional locoregional surgical procedure; (2) patient primary therapy outcome success and overall survival information was recorded; and (3) patient tumor samples received RNA sequencing (RNA-seq) and/or miRNA sequencing (miRNA-seq). The following four levels of primary therapy outcome were assessed: complete remission/response (CR), partial remission/response (PR), stable disease (SD), and progressive disease (PD). Patients with CR and PR were classified into the radiotherapy-sensitive group, while patients with SD and PD were classified into the radiotherapy-resistant group. Eighty-seven NSCLC patients with RNA-seq and 83 NSCLC patients with miRNA-seq met the requirements (Table 1). Moreover, autophagy-related genes (ARGs) and gene sets (ARGSs) were acquired from the Gene Ontology (GO) resource^2^. The study was then extended to thoroughly investigate the clinical significance and regulatory mechanism of autophagy in the radiotherapy response of NSCLC patients. Differential expression of RNAs and miRNAs was analyzed, and the score of ARGSs was calculated. Clinical correlation and immune microenvironment analysis were then performed at both the gene and gene set levels. Moreover, radiotherapy sensitivity- and overall survival (OS)-related risk signatures were constructed for prognostic prediction. A circRNA-miRNA-ARmRNA-ARGS network was constructed following target prediction and correlation analysis.

**TABLE 1:** Patients’ characteristics.

Patients with RNA-seq data (*N*=87)		Patients with miRNA-seq data (*N*=83)	
Variables	No. (% or range)	Variables	No. (% or range)
**Age**		**Age**	
≤ 60	35 (0.40)	≤ 60	33 (0.40)
> 60	52 (0.60)	> 60	50 (0.60)
**Gender**		**Gender**	
Female	38 (43.7)	Female	36 (0.43)
Male	49 (56.3)	Male	47 (0.57)
**Race**		**Race**	
Asian	3 (0.03)	Asian	3 (0.04)
White	66 (0.08)	White	64 (0.77)
Black	7 (0.13)	Black	7 (0.08)
Not reported	11 (0.76)	Not reported	9 (0.11)
**Year of diagnosis**		**Year of diagnosis**	
Before 2004	18 (0.21)	Before 2004	18 (0.22)
2005∼2008	24 (0.28)	2005∼2008	21 (0.25)
2009∼2013	45 (0.52)	2009∼2013	44 (0.53)
**Histology**		**Histology**	
AD^1^	49 (0.56)	AD	48 (0.58)
SCC^2^	38 (0.44)	SCC	35 (0.42)
**Primary site**		**Primary site**	
Lower lobe	27 (0.31)	Lower lobe	25 (0.30)
Upper lobe	54 (0.62)	Upper lobe	52 (0.63)
Not reported	6 (0.07)	Not reported	6 (0.07)
**TNM edition number**		**TNM edition number**	
Before 5th	9 (0.10)	Before 5th	9 (0.11)
6th	41 (0.47)	6th	38 (0.46)
7th	31 (0.36)	7th	31 (0.37)
Not reported	6 (0.07)	Not reported	5 (0.06)
**T stage**		**T stage**	
T1	18 (0.21)	T1	15 (0.18)
T2	48 (0.55)	T2	47 (0.57)
T3	16 (0.18)	T3	16 (0.19)
T4	4 (0.05)	T4	4 (0.05)
Tx	1 (0.01)	Tx	1 (0.01)
**N stage**		**N stage**	
N0	34 (0.39)	N0	32 (0.39)
N1	20 (0.23)	N1	19 (0.23)
N2	27 (0.31)	N2	27 (0.33)
N3	3 (0.03)	N3	2 (0.02)
Nx	3 (0.03)	Nx	3 (0.04)
**M stage**		**M stage**	
M0	65 (0.75)	M0	62 (0.75)
M1	6 (0.07)	M1	5 (0.06)
Mx	16 (0.18)	Mx	16 (0.19)
**Stage**		**Stage**	
Stage I	21 (0.24)	Stage I	20 (0.24)
Stage II	23 (0.26)	Stage II	22 (0.27)
Stage III	37 (0.43)	Stage III	36 (0.43)
Stage IV	6 (0.07)	Stage IV	5 (0.06)
**Radiotherapy sensitivity**		**Radiotherapy sensitivity**	
Sensitivity	48 (0.55)	Sensitivity	45 (0.46)
Resistance	39 (0.45)	Resistance	38 (0.54)

*^1^AD: adenocarcinoma;*

*^2^SCC: squamous cell carcinoma.*

### Identification and Extraction of Autophagy-Related Genes and Gene Sets

A total of 537 unduplicated autophagy-related genes (ARGs) were extracted from GO:0006914, and 9 autophagy-related gene sets (ARGSs) were identified (Supplementary Figure 1 and Supplementary Table 1). The genes were related to the following types of autophagy: 78 genes were related to autophagy of mitochondrion (GO:0000422); 8 genes were related to autophagy of peroxisome (GO:0030242); 18 genes were related to chaperone-mediated autophagy (GO:0061684); 5 genes were related to late endosomal microautophagy (GO:0061738); 308 genes were related to macroautophagy (GO:0016236); 17 genes were related to autophagy of nucleus (GO:0044804); 7 genes were related to lysosomal microautophagy (GO:0016237); 336 genes were related to regulation of autophagy (GO:0010506); and 9 genes were related to modulation by symbiont of host autophagy (GO:0075071).

### Evaluation of the Immune Characteristics of Tumor Microenvironment (TME)

The immune characteristics of TME included the expression level of immune checkpoint inhibitors (ICIs), infiltration of tumor-infiltrating immune cells (TIICs), and activity of the cancer immune cycle. Overall, 20 ICIs (HAVCR2, CD274, CD86, LAG3, LAIR1, PVR, IDO1, CD80, CTLA4, SNCA, TIGIT, CD200R1, CEACAM1, CD276, CD200, KIR3DL1, BTLA, ADORA2A, LGALS3, and VTCN1) with therapeutic potential (Noam et al., 2018) were identified in our study. The infiltration levels of 28 tumor-infiltrating immune cells (activated B cells, activated CD4 T cells, activated CD8 T cells, activated dendritic cells, CD56 bright NK cells, CD56 dim NK cells, central memory CD4 T cells, central memory CD8 T cells, effector memory CD4 T cells, effector memory CD8 T cells, eosinophil cells, gamma delta T cells, immature B cells, immature dendritic cells, macrophages, mast cells, MDSCs, memory B cells, monocytes, NK cells, NK T cells, neutrophils, plasmacytoid dendritic cells, regulatory T cells, T follicular helper cells, TH1 cells, TH17 cells, and TH2 cells) (Charoentong et al., 2017) were considered. The cancer immune cycle mainly comprises the following seven steps: release of cancer cell antigens (Step 1); cancer antigen presentation (Step 2); priming and activation (Step 3); trafficking of immune cells to tumors (Step 4); infiltration of immune cells into tumors (Step 5); recognition of cancer cells by T cells (Step 6); and killing of cancer cells (Step 7) (Chen and Mellman, 2013). The activity of TIICs and the cancer immune cycle were evaluated by calculating marker gene set scores based on the gene expression of individual samples.

### Screening of Differentially Expressed Genes

Available RNA sequencing (RNA-seq) and miRNA sequencing (miRNA-seq) data were downloaded. We transformed miRNA-seq names into human mature miRNA names using the miRBase version 22.0 database. We then applied DESeq2, edgeR, and limma/voom to identify differentially expressed mRNAs (DEmRNAs) and miRNAs (DEmiRNAs). The criteria for determining differential DEmRNAs and DEmiRNAs were set with an adjusted *p*-value < 0.05 and | log fold change (FC)| > mean ± standard deviation (sd). We determined the common DEmRNAs and DEmiRNAs by utilizing the VennDiagram R package (Chen and Boutros, 2011). Volcano plots visually displaying the distribution of DEmRNAs and DEmiRNAs were generated using ggpubr R packages, and heatmaps describing the expression of differentially expressed autophagy-related mRNAs (DEARmRNAs) and miRNAs (DEmiRNAs) were generated utilizing the pheatmap R package.

### Establishment of Specific Risk Signatures

We extracted the DEmRNAs and DEmiRNAs expression profiles collected from NSCLC patients receiving radiotherapy with prognostic information. Differential analysis by Student’s *t*-test was conducted to compare the radiotherapy-resistant and radiotherapy-sensitive groups, while the overall survival (OS) difference was calculated by the log-rank test and described by the K-M curve. The significant variables were included in a logistic or Cox regression model. Finally, we generated an autophagy-related radiosensitivity risk signature (ARRS) and an autophagy-related OS risk signature (AROS) for each sample using the following equation: ARRS or AROS = ∑_*i*_ *Cofficient*(*RNAi*) × *Expression*(*RNAi*). The receiver operating characteristic (ROC) curves with risk score against radiosensitivity and survival status were generated using ROCit and survivalROC/timeROC R packages (Blanche, 2015), respectively. Based on the mean as a cutoff point, patients were divided into high- and low-risk groups. Student’s *t*-test and log-rank test were used in univariate differential analysis, while multivariate logistic and Cox regression were used in independent predictor tests.

### Construction of the circRNA-miRNA-mRNA-ARGS Network

DEARmRNAs were the key module in the ceRNA network. MiRNAs targeting DEARmRNAs were predicted by miRWalk 3.0^3^ (Dweep et al., 2011). These miRNAs were intersected with DEmiRNAs to obtain the miRNA module. The circRNA sponges of the miRNA modules were obtained by circBank^4^ (Liu et al., 2019). CircRNAs sponging more than one candidate miRNAs were included in the circRNA module. Correlation analysis was then performed between the ARmRNA module and miRNA module in the total population of the TCGA NSCLC project to obtain the negatively correlated ARmRNAs and miRNAs. The targeted ARGSs of ARmRNAs and circRNA sponges of miRNAs were added to construct the final regulatory network. The R package ggalluvial and Cytoscape (Shannon et al., 2003) were used to visualize the ceRNA and circRNA-miRNA-ARmRNA-ARGS network.

### Additional Bioinformatics and Statistical Analyses

R software 4.0.4^5^, GraphPad Prism 9.0 (GraphPad Software Inc., San Diego, CA, United States), and Cytoscape 3.8.2^6^ were used to analyze and visualize the data. The scores of gene sets (ARGSs, immune characteristics of the tumor microenvironment) in each sample were quantified via both single-sample gene set enrichment analysis (ssGSEA) and the gene set variation analysis (GSVA) algorithm based on the bulk RNA-seq data using the GSVA R package (Hnzelmann et al., 2013; Supplementary Table 2). We used the chi-square test for correlation analysis between categorical variables, and Pearson correlation coefficients for correlation analysis between continuous variables. A *p* value < 0.05 was considered statistically significant.

## Results

### Clinical Significance of Autophagy-Related Gene Sets in NSCLC Patients Treated With Radiotherapy

**FIGURE 2:**
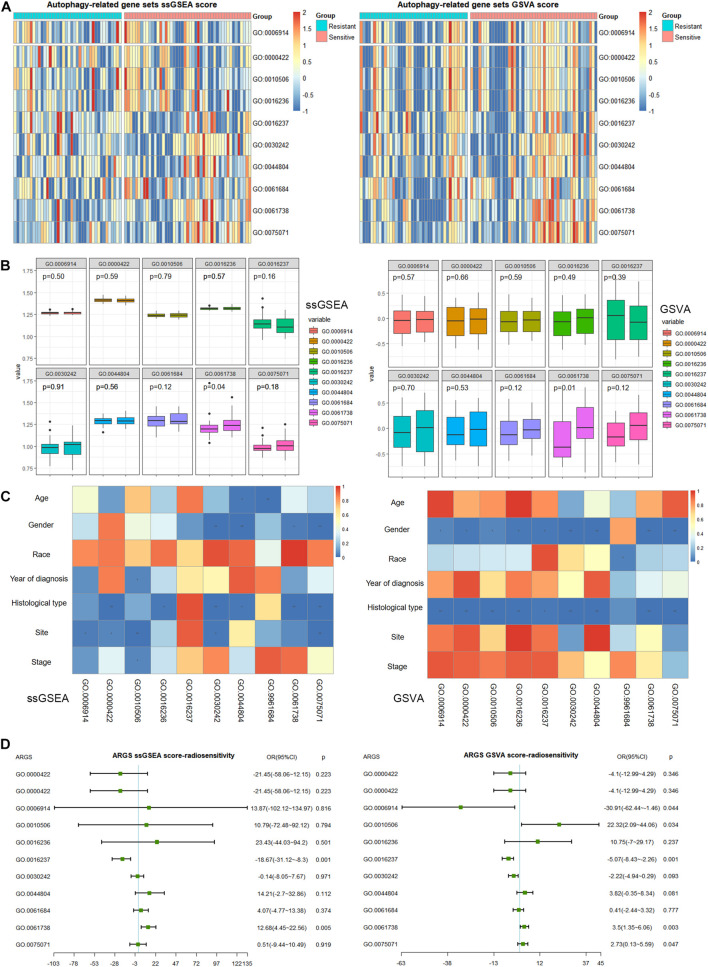
Clinical correlation of autophagy-related gene sets (ARGSs) score by ssGSEA and GSVA methods. **(A)** The distribution of ARGSs score in radiotherapy resistant and sensitive groups. **(B)** The differential analysis of ARGSs score between radiotherapy resistant and sensitive groups. The left is radiotherapy resistant group while the right is radiotherapy sensitive group. **(C)** The correlation analysis of ARGSs and clinicopathologic factors. **(D)** Multivariate logistic regression analysis of ARGSs with radiotherapy responses.

We plotted heatmaps to describe the distribution of ARGS scores by ssGSEA or GSVA (Figure 2A) and performed differential analysis between the radiotherapy-resistant and radiotherapy-sensitive groups. Late endosomal microautophagy (GO:0061738) was identified as significant by both methods (Figure 2B). The correlation between clinicopathological factors and ARGS was evaluated (Figure 2C). The consistent results of ssGSEA and GSVA score showed that autophagy of mitochondrion (GO:0000422) and macroautophagy (GO:0016236) were discriminatory for different histological types, while autophagy of peroxisome (GO:0030242), autophagy of nucleus (GO:0044804), late endosomal microautophagy (GO:0061738), and symbiont of host autophagy (GO:0075071) were discriminatory for patient gender and histological type. Multivariate logistic regression analysis demonstrated that lysosomal microautophagy (GO:0016237) and late endosomal microautophagy (GO:0061738) were independent risk factors for radiotherapy sensitivity (Figure 2D), while no ARGSs were associated with OS (Supplementary Figure 2). These findings demonstrated the clinical significance of autophagy or selective types of autophagy in NSCLC patients receiving radiotherapy.

### Correlation Analysis of ARGSs and Immune Microenvironment Characteristics in NSCLC Patients Receiving Radiotherapy

With regard to immune microenvironment characteristics (Supplementary Figure 3A and Supplementary Table 3), autophagy (GO:0006914), regulation of autophagy (GO:0010506), macroautophagy (GO:0016236), and autophagy of peroxisome (GO:0030242) were related to infiltration of central memory CD8 T cells and gamma delta T cells (Supplementary Figure 3B). In addition, macroautophagy (GO:0016236) was related to infiltration of CD56 bright NK cells, and symbiont of host autophagy (GO:0075071) was related to central memory CD4 T cells. Regarding the immune cycle, only autophagy of peroxisome (GO:0030242) correlated with the trafficking of monocytes to tumors (Step 4) (Supplementary Figure 3C). Finally, and most importantly, we evaluated the association with ICIs (Supplementary Figure 3D). Autophagy (GO:0006914) was associated with expression levels of ADORA2A, CD200R1, CD274, CD80, CD86, HAVCR2, LAIR1, LGALS3, and TIGIT; autophagy of mitochondrion (GO:0000422) was correlated with CD276 and SNCA; regulation of autophagy (GO:0010506) was correlated with ADORA2A, CD200R1, CD274, CD80, CD86, HAVCR2, LAIR1, and TIGIT; macroautophagy (GO:0016236) was correlated with CD80, CD86, HAVCR2, LAG3, and LAIR1; autophagy of peroxisome (GO:0030242) was correlated with CD276 and SNCA; chaperone-mediated autophagy (GO:0061684) was correlated with CD200R1, CD80, CD86, HAVCR2, LAG3, and LAIR1; and late endosomal microautophagy (GO:0061738) and symbiont of host autophagy (GO:0075071) were correlated with SNCA.

### Identification of Differentially Expressed Autophagy-Related mRNAs (DEARmRNAs) and miRNAs (DEmiRNAs) Associated With Radiotherapy Sensitivity in NSCLC Patients

**FIGURE 3:**
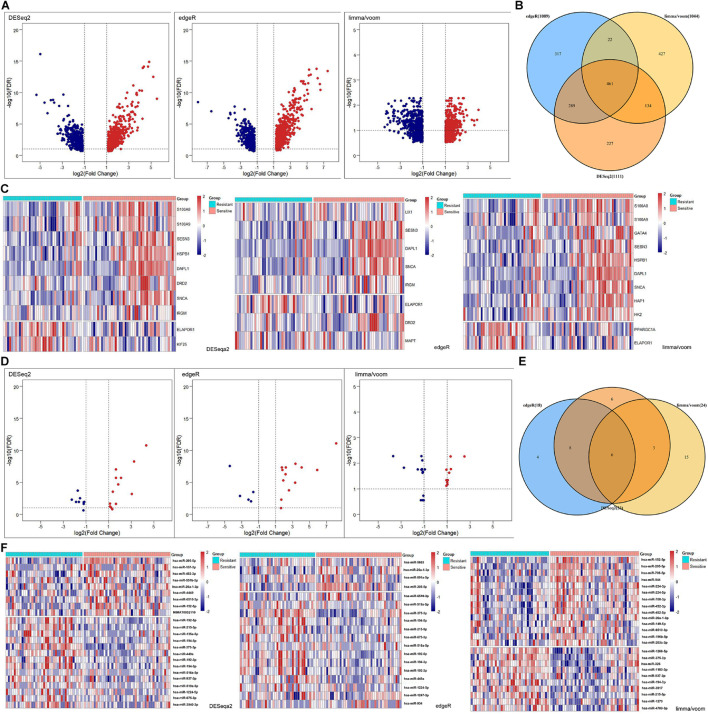
Differentially expressed (DE) analysis of mRNAs and miRNAs between rediotherapy resistant and sensitive groups. **(A)** Volcano plots of DEmRNAs by DESeq2, edgeR, and limma/voom. **(B)** Venn diagram of common DEmRNAs. **(C)** Heatmaps of DEARmRNAs. **(D)** Volcano plots of DEmiRNAs by DESeq2, edgeR, and limma/voom. **(E)** Venn diagram of common DEmiRNAs. **(F)** Heatmaps of DEmiRNAs. Red represents upregulated genes and blue indicates downregulated genes.

In addition to the levels of gene sets, we explored the clinical significance of autophagy at the gene level. The DEmRNAs were identified from the radiotherapy-sensitive group compared to the radiotherapy-resistant group by DESeq2, edgeR, and limma/voom seperately (Figure 3A). In total, 461 DEmRNAs (235 upregulated and 226 downregulated) were found by intersection of these three methods (Figure 3B). Then, DEARmRNAs were recognized from DEmRNAs (Figure 3C) and one downregulated gene (ELAPOR1), three upregulated genes (SNCA, SESN3, and DAPL1) were the consistent results. The same methods were used in DEmiRNA screening (Figure 3D), which identified 3 upregulated (hsa-miR-205-5p, hsa-miR-26a-1-3p, and hsa-miR-6510-3p) and 3 downregulated (hsa-miR-194-3p, hsa-miR-215-5p, and hsa-miR-375-3p) DEmiRNAs by intersection of DESeq2, edgeR, and limma/voom (Figures 3E,F).

### Validation of the Prognostic Value of DEARmRNAs and DEmiRNAs in NSCLC Patients Receiving Radiotherapy

**FIGURE 4:**
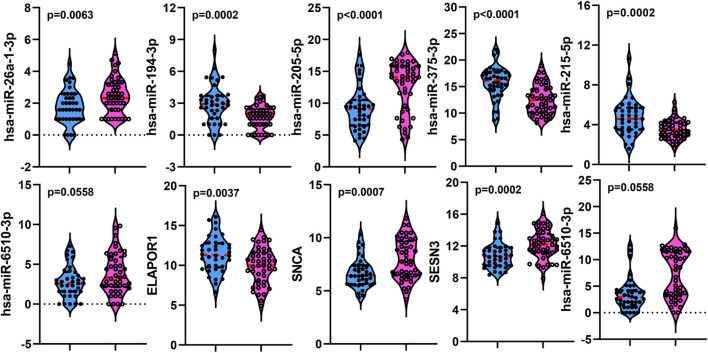
The distribution of differentially expressed miRNAs and mRNAs between rediotherapy resistant and sensitive groups. Blue represents radiotherapy resistant group and pink indicates radiotherapy sensitive group.

**FIGURE 5:**
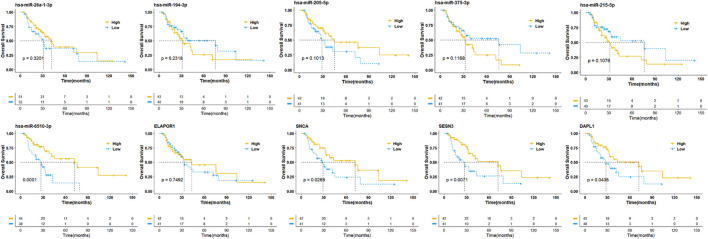
Overall survival analysis of differentially expressed miRNAs and mRNAs. The high- and low-expression values of four autophagy-related mRNAs (ARmRNAs) and six miRNAs were compared by Kaplan-Meier survival curve for NSCLC patients. The median survival time were indicated by dashed line.

To establish crucial miRNAs and ARmRNAs with prognostic value in NSCLC patients receiving radiotherapy, we first verified the differential expression of mRNAs and miRNAs between radiotherapy-sensitive and radiotherapy-resistant groups. Our results showed that hsa-miR-194-3p, hsa-miR-215-5p, hsa-miR-375-3p, and ELAPOR1 were upregulated in the radiotherapy-resistant group, while hsa-miR-205-5p, hsa-miR-26a-1-3p, SESN3, SNCA, and DAPL1 were upregulated in the radiotherapy-sensitive group (Figure 4). To determine whether these DERNAs are associated with the long-term prognosis of NSCLC patients treated with radiotherapy, we generated Kaplan-Meier curves to analyze differences in OS. We found that SNCA, SESN3, and DAPL1 were related to the OS of NSCLC patients (Figure 5).

### Establishment of the ARmRNA/miRNA Signature to Predict Prognosis in NSCLC Patients Receiving Radiotherapy

**FIGURE 6:**
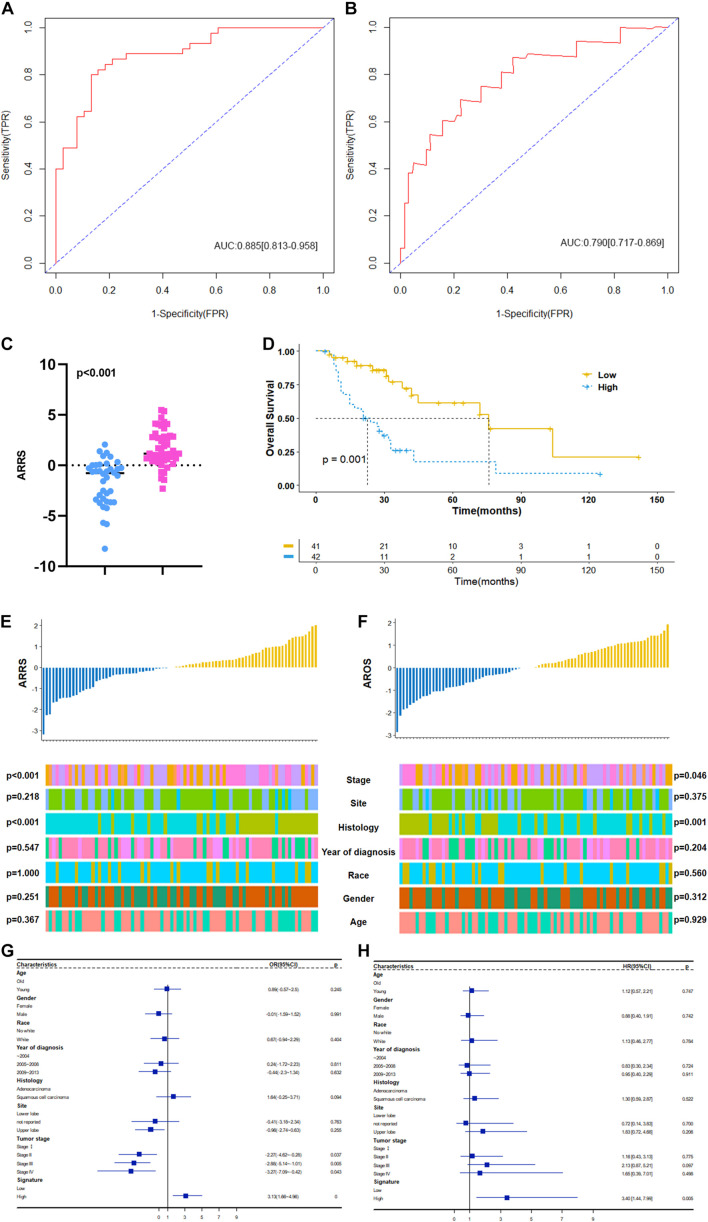
Construction and clinical correlation analysis of autophagy-related prognostic risk signature. **(A)** The receive operator curve (ROC) analysis for autophagy-related radiosensitivity risk signature (ARRS). AUC: Area Under Curve, FPR: false positive rate, TPR: true positive rate. **(B)** The ROC analysis for autophagy-related overall survival risk signature (AROS). **(C)** The distribution of ARRS between rediotherapy resistant and sensitive groups. Blue represents radiotherapy resistant group and pink indicates radiotherapy sensitive groups. **(D)** The Kaplan-Meier survival curve grouping by high- and low- AROS. The median survival time were indicated by dashed line. **(E)** The correlation analysis of ARRSs and clinicopathologic factors. **(F)** The correlation analysis of AROSs and clinicopathologic factors. **(G)** Multivariate logistic regression analysis of ARRSs with radiotherapy sensitivity. **(H)** Multivariate Cox regression analysis of AROSs with overall survival.

Based on the above results, we first established a 9 ARmRNA/miRNA signature by multivariate logistic regression to predict the radiosensitivity of NSCLC patients, and the score for each patient was calculated as follows: ARRS = 1.543103 −0.002191^∗^hsa-miR-205-5p −0.500703^∗^hsa-miR-215-5p +0.776517^∗^hsa-miR-26a-1-3p −0.300282^∗^hsa-miR-194-3p −0.041439^∗^hsa-miR-375-3p −0.394497^∗^ELAPOR1 −0.053145^∗^SNCA +0.331343^∗^SESN3 +0.181778^∗^DAPL1. The ROC curve was generated, and the estimated AUC was 0.885 with a 95% CI of 0.813–0.958 (Figure 6A). The ARRS discriminated the radiotherapy-sensitive group from the radiotherapy-resistant group (*p* < 0.001) by higher ARRS score (Figure 6C) and related to histology and stage (Figure 6E). Besides, ARRS could serve as an independent radiotherapy sensitivity predictor for NSCLC and high ARRS score patients are more likely to get better radiotherapy sensitivity (OR:3.13[95%CI:1.66–4.96], *p* < 0.001) (Figure 6G).

Moreover, a 3 ARmRNA signature was generated by multivariate Cox regression to predict the OS of NSCLC patients, and the score for each patient was calculated as follows: AROS = −0.19011^∗^hsa-miR-6510-3p −0.18664^∗^SNCA −0.14049^∗^SESN3 +0.06797^∗^DAPL1. The ROC curve was generated, and the estimated AUC was 0.790, with a 95% CI of 0.717–0.869 (Figure 6B). The K-M curves were different between the high- and low-AROS groups (*p* < 0.001) (Figure 6D), and multivariate Cox regression revealed that AROS served as an independent predictor of OS for NSCLC patients who scored higher AROS with shorter OS (HR:3.40[95%CI:1.44–7.99], *p* = 0.005) (Figure 6H). The AROS was also related to histology and stage (Figure 6F).

### Landscape of Immune Microenvironment Characteristics Associated With the ARmRNA/miRNA Signature

**FIGURE 7:**
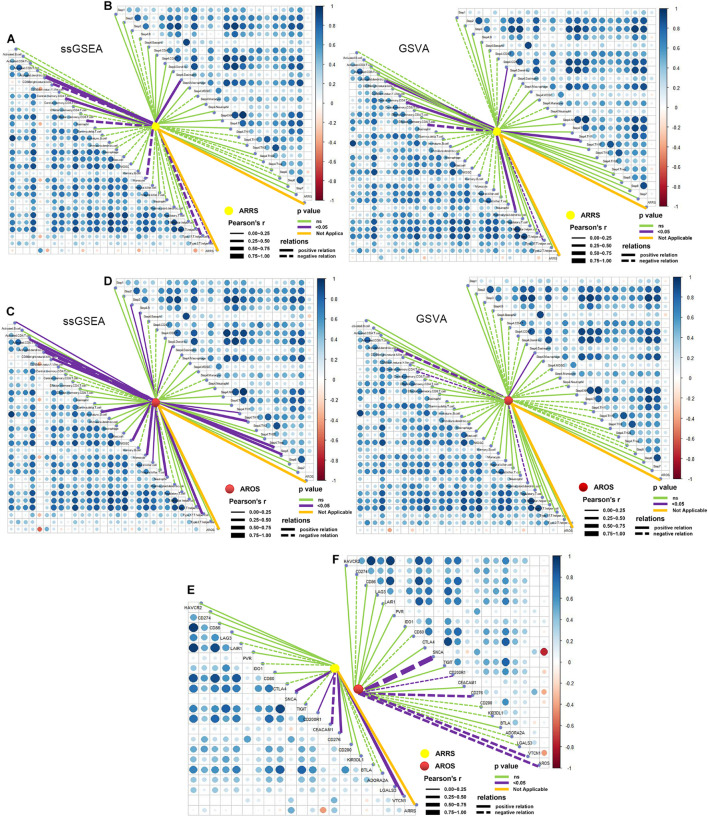
Role of autophagy-related prognostic risk signature in predicting immune phenotypes. **(A)** Correlations between ARRS and infiltration levels of tumor-associated immune cells. **(B)** Correlations between ARRS and immune cycle. **(C)** Correlations between AROS and infiltration levels of tumor-associated immune cells. **(D)** Correlations between AROS and immune cycle. **(E)** Correlations between ARRS and immune checkpoint inhibitors. **(F)** Correlations between AROS and immune checkpoint inhibitors.

The two prognosis-related signatures (ARRS and AROS) were then estimated for immune microenvironment characteristics. ARRS positively correlated with the infiltration of CD56 bright NK cells and central memory CD8 T cells but negatively correlated with eosinophils and type 17 T helper cells (Figure 7A), while AROS negatively correlated with the infiltration of CD56 bright NK cells in both methods (Figure 7C). In terms of the immune cycle, ARRS was positively correlated with the trafficking of eosinophil cells to tumors (Step 4) (Figure 7B), while AROS was positively correlated with the trafficking of TH1 cells to tumors (Step 4) (Figure 7D) in both methods. Finally, we also investigated the relationship between ARRS or AROS and the expression levels of immune checkpoint molecules. Low ARRS indicated low expression of SNCA, CD200R1, CD276, LGALS3, and VTCN1 but high expression of CEACAM1 (Figure 7E), while low AROS represented high expression of SNCA, CD200R1, CD276, and VTCN1 (Figure 7F).

### Construction of the circRNA-miRNA-ARmRNA-ARGS Network to Regulate the Radiation Sensitivity of NSCLC

**FIGURE 8:**
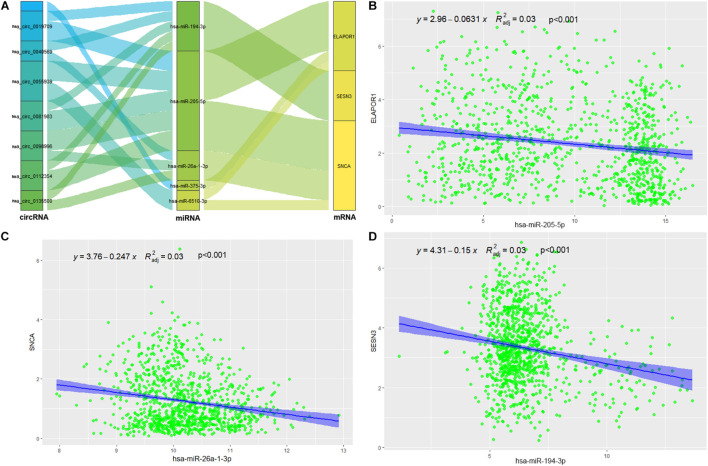
Construction and correlation analysis of the ceRNA network. **(A)** The alluvial diagram of regulatory network of ceRNA. **(B)** Correlation analysis between has-miR-205-5p and ELAPOR1. **(C)** Correlation analysis between has-miR-26-1-3p and SNCA. **(D)** Correlation analysis between has-miR-194-3p and SESN3.

**FIGURE 9:**
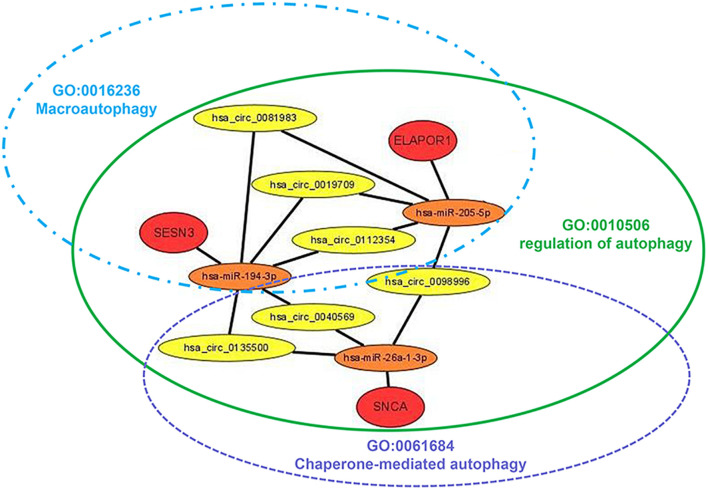
Diagram of the schematics of the circRNA-miRNA-ARmRNA-ARGS network. Red circles represent high-risk genes; orange circles represent their, respectively, identified regulatory miRNAs; yellow circles represent sponge circRNAs; green, blue, and purple ellipse represent corresponding ARGSs.

The targeted miRNAs of DEARmRNAs were predicted by miRWalk (Supplementary Table 4) and intersected with DEmiRNAs. We obtained three candidate ARmRNAs (ELAPOR1, SESN3, and SNCA) and 5 miRNAs (hsa-miR-26a-1-3p, hsa-miR-6510-3p, hsa-miR-205-5p, hsa-miR-375-3p, and hsa-miR-194-3p) for network construction (Figure 8A). Considering the conventionally negative correlation between mRNAs and miRNAs in regulatory relationships, we used the total population of TCGA NSCLC project for correlation analysis between these three ARmRNAs and their targeted miRNAs. After secondary screening, three miRNA/ARmRNA axes were recognized, namely, miR-205-5p/ELAPOR1, miR-26a-1-3p/SNCA, and miR-194-3p/SESN3 (Figures 8B–D). We then used circBank to identify the circRNAs targeting these three miRNAs (Supplementary Table 4). To enhance the affinity between circRNAs and the ceRNA network, we sought candidate circRNAs targeting two or more miRNAs. Finally, six circRNAs (hsa_circ_0019709, hsa_circ_0081983, hsa_circ_0112354, hsa_circ_0040569, hsa_circ_0135500, and hsa_circ_0098966) were identified. Finally, ARmRNA-associated ARGSs were added to form a complete regulatory network (Figure 9). All three ARGs (ELAPOR1, SNCA, and SESN3) participate in the regulation of autophagy. ELAPOR1 and SESN3 are involved in macroautophagy, and SNCA participates in chaperone-mediated autophagy.

## Discussion

Although autophagy can be non-specific, there are many selective types of autophagy (Klionsky et al., 2016). For a more detailed exploration of the role of autophagy in radiotherapy responses, we referred to the GO source to identify 9 ARGSs in the present study. The differential analysis of ARGS scores revealed that late endosomal microautophagy was distinct between the radiotherapy-sensitive and radiotherapy-resistant groups. Endosomal microautophagy requires endosomal-sorting complex systems for lysosome or endosome delivery and selectively degrades KFERQ-containing proteins recognized by HSC70 (Zheng et al., 2019). Microautophagy is the least studied form of autophagy with a largely unclear cargo delivery process (Zheng et al., 2019). The significance of endosomal microautophagy in the radiotherapy sensitivity of NSCLC patients was first proposed in our study; the intrinsic mechanism is worth pursuing in the future.

Competing endogenous RNAs (ceRNAs) are transcripts that competitively bind to shared miRNAs and act as miRNA sponges to modulate each other at the posttranscriptional level (Qi et al., 2015). With the development of high-throughput sequencing technology, abundant circRNAs have been identified and have become the focus in the ceRNA family due to the abundance of conserved miRNA response elements (MREs) (Zhong et al., 2018). Previous research has demonstrated that one of the most important mechanisms of circRNAs is their action on ceRNAs. For example, circRNA hsa_circ_100395 has been demonstrated to inhibit lung cancer progression by regulating the miR-1228/TCG21 pathway (Chen et al., 2018), while circRNA_101237 promotes NSCLC progression by regulating the miR-490-3p/MAPK1 axis (Zhang Z. Y. et al., 2020). Moreover, Jin et al. (2020) revealed potential prognostic biomarkers for radiotherapies with X-rays and carbon ions in NSCLC by integrating analysis of the circRNA-miRNA-mRNA network. Overall, the role of the circRNA-miRNA-mRNA network in the radiotherapy sensitivity of NSCLC still needs further research. In our study, after generating three miRNA-ARmRNA axes (miR-194-3p/SESN3, miR-205-5p/ELAPOR1, and miR-26a-1-3p/SNCA), we obtained six circRNAs (hsa_circ_0019709, hsa_circ_0040569, hsa_circ_0081983, hsa_circ_0098996, hsa_circ_0112354, hsa_circ_0135500) that intersected these three axes and constructed a circRNA-miRNA-ARmRNA network. It is worth noting that these ARmRNAs were contained in three ARGSs, namely, regulation of autophagy, macroautophagy, and chaperone-mediated autophagy. Macroautophagy refers to the process of autophagosomes formation and fusion with late endosomes or lysosomes to form amphisomes or autolysosomes, which are the canonical and well-known participants in the autophagy process (Zheng et al., 2019). Chaperone-mediated autophagy (CMA) is another vital type of selective autophagy which selectively degrades cytosolic proteins recognized by a specific chaperone in lysosomes (Zheng et al., 2019). CMA does not rely on vesicles or membrane invagination to deliver targeted substrates and degrades 30% of cytosolic proteins. SESN3 encodes a member of the sestrin family of stress-induced proteins, which may contribute to the positive regulation of macroautophagy (Pascale et al., 2011). ELAPOR1 is an endosome-lysosome-associated apoptosis and autophagy regulator, and it may protect cells from cell death by upregulating the autophagy pathway (Deng et al., 2010). SNCA is a member of the synuclein family and negatively regulates CMA (Alvarez-Erviti et al., 2010). In summary, our study utilized circRNA-miRNA-ARmRNA network analysis to investigate the subtypes of autophagy.

With the general success of immune checkpoint inhibitor antibodies and cell-based treatments, the age of immunotherapy has arrived, which raises the question of how autophagy interacts with the immune microenvironment and contributes to cancer treatments (Li et al., 2017; Jiang et al., 2019). It remains unclear whether autophagy inhibition impairs systematic immunity. Some evidence has shown that autophagy maintains the survival of memory T cells (Puleston et al., 2014) and promotes self-renewal of B1 cells (Clarke et al., 2018), while other evidence has shown that autophagy inhibition does not impair T cell function in preclinical models of melanoma and breast cancer, including chemotherapy-treated cells (Starobinets et al., 2016). Although a greater understanding of the role of autophagy in tumor immunity is emerging, the distinction between canonical autophagy and types of selective autophagy needs to be considered. Correlation analysis of ARGSs and ICI expression levels, immune cell infiltration, and the immune cycle was conducted in our work. We found that autophagy was related to the expression levels of many ICIs and the infiltration of central memory CD8 T cells and gamma delta T cells, while peroxisome autophagy correlated with the trafficking of monocytes to tumors. Though more extensive experiments are needed to confirm this model, these results support that autophagy levels are in tune with the immune microenvironment and have the potential to contribute to monitoring and improving immunotherapy in NSCLC patients.

Despite the tremendous development of radiation technology, tumor control and survival in NSCLC patients have not substantially improved. Individual heterogeneity partly explains this. Some patients may benefit from specific treatments while others require more aggressive treatments. To improve clinical outcomes and avoid excessive medical treatment, patients should be classified into cohorts according to differences in disease susceptibility, prognosis, and likely treatment response rates (Meehan et al., 2020). Additionally, the incorporation of molecular analysis and other patient information into the prevention, investigation, and treatment of diseases is an important aspect of precision medicine (Penet et al., 2014). Some efforts have been made to identify biomarkers that could be applied to tailor radiotherapy sensitivity to individual molecular characteristics of patient tissue. Salem et al. (2017) reported a blood biomarker panel containing interleukin (IL)-1b, neutrophil count, and cytokeratin-19 antigen to predict lung cancer radiotherapy response. Saito et al. (2014) constructed a three-microRNA signature to predict responses to platinum-based doublet chemotherapy in patients with lung adenocarcinoma. Liu et al. (2019) identified a miRNA signature by an *in vitro* system to assess radiosensitivity for head and neck squamous cell carcinomas and validated this signature using the TCGA database (Ning et al., 2015). These studies indicate that radiotherapy sensitivity should be considered before designing the treatment plan. Furthermore, short-term radiotherapy response does not always equate to long-term treatment outcomes. Hence, we also established an OS-related signature (AROS) beyond a radiotherapy sensitivity predictive signature (ARRS). The differential expression analysis and autophagy-related gene selection provided strong background support.

With the advent of immunotherapy, the interaction of radiotherapy and the immune system has gained widespread interest, and this interaction has been increasingly reported in NSCLC (Herter-Sprie et al., 2016). Radiotherapy has been demonstrated to promote tumor cell death and enhance antitumor immune responses by converting poorly immunogenic tumors into more highly immunogenic ones, not only through immunogenic cell death (ICD) but also through the modification of the characteristics of key immune cells within the tumor microenvironment (Keam et al., 2020). However, radiotherapy may be a double-edged sword; it induces activation and infiltration of T cells to the tumor bed, but it also triggers migration of immunosuppressive cells and upregulates inhibitory ligands and receptors (Keam et al., 2020). To improve the beneficial effects and reduce the risks, the biological responses and toxicities of radiation and drugs should be accurately modeled. However, the combination and the optimal timing, dose, or schedules of radiotherapy and immunotherapy are still controversial (Aliru et al., 2018). In addition to investigating the molecular features of patients’ responses to radiotherapy, we also described their tumor microenvironment by the bulk RNA-seq results in the present study. Autophagy-related risk scores predict not only radiotherapy sensitivity and OS but also the landscape of ICIs, immune cell infiltration, and immune cycle activation. These signature models may aid treatment decision making with consideration of concurrent radiotherapy and immunotherapy.

There were several limitations to this study. First, due to the incompleteness of primary therapy outcome success data in TCGA, fewer than 90 patients met our inclusion requirements. In addition, the lack of an index may render an inaccurate interpretation. Second, the prognostic signature was not validated because of the rarity of data sets recording radiotherapy responses in NSCLC patients. Third, although a potential regulatory mechanism has been constructed, no experimental support was provided. To ameliorate the limitations described above, single institution or multicenter clinical retrospective or prospective study should be conducted to verify the predictive value of prognostic signatures, and experiments should be rigorously designed to demonstrate the regulatory network of NSCLC.

In conclusion, we examined the role of autophagy-related genes (ARGs) and gene sets (ARGSs) in the radiotherapy response of NSCLC patients by mining public data. First, we verified the clinical significance of autophagy in the radiotherapy response of NSCLC patients by analyzing the correlation between ARGs or ARGSs and clinicopathologic factors, prognosis, and the immune microenvironment. In addition, the circRNA-miRNA-ARmRNA-ARGS network was constructed to predict the regulatory mechanisms underlying the radiation response of NSCLC. In summary, our work provided useful information to introduce potential molecular classifications and regulatory mechanisms into radiotherapy short- and long-term responses of NSCLC patients.

## Data Availability Statement

The original contributions presented in the study are included in the article/Supplementary Material, further inquiries can be directed to the corresponding authors.

## Author Contributions

LF, BL, and LS conceived and designed the whole project. LF, ZL, and LS analyzed the data and wrote the manuscript. BL helped the data discussion. All authors read and approved the final manuscript.

## Conflict of Interest

ZL is employed by Shandong Yidian Gene Technology Co., Ltd. The remaining authors declare that the research was conducted in the absence of any commercial or financial relationships that could be constructed as a potential conflict of interest.

## Publisher’s Note

All claims expressed in this article are solely those of the authors and do not necessarily represent those of their affiliated organizations, or those of the publisher, the editors and the reviewers. Any product that may be evaluated in this article, or claim that may be made by its manufacturer, is not guaranteed or endorsed by the publisher.

## Footnotes

1
https://xena.ucsc.edu/

2
http://geneontology.org/

3
http://mirwalk.umm.uni-heidelberg.de/

4
http://www.circbank.cn/

5
http://www.r-project.org

6
https://cytoscape.org/

## Supplementary Material

Supplementary Figure 1 Identification of autophagy-related gene sets (ARGSs).

Supplementary Figure 2 Multivariate Cox regression analysis of ARGSs with overall survival.

Supplementary Figure 3 Role of ARGS in predicting immune phenotypes. **(A)** The content of tumor immune microenvironment. **(B)** Correlations between ARGSs and infiltration levels of tumor-associated immune cells. **(C)** Correlations between ARGSs and immune cycle. **(D)** Correlations between ARGSs and immune checkpoint inhibitors. Upper left of **(B–D)** represent the *r* value while upper right represent *p* value by calculating ARGSs ssGSEA score; lower left represent *r* value while lower right represent *p* value by calculating ARGSs GSVA score.

Supplementary Figure 4 Correlation analysis between other predicted miRNAs and ARmRNAs.
