# Supplementary figures and images for: Identification of Autophagy Related circRNA-miRNA-mRNA-Subtypes Network With Radiotherapy Responses and Tumor Immune Microenvironment in Non-small Cell Lung Cancer
Source: Front Genet. 2021 Sep 9;12:730003. doi: 10.3389/fgene.2021.730003 (PMC8458766; doi:10.3389/fgene.2021.730003)

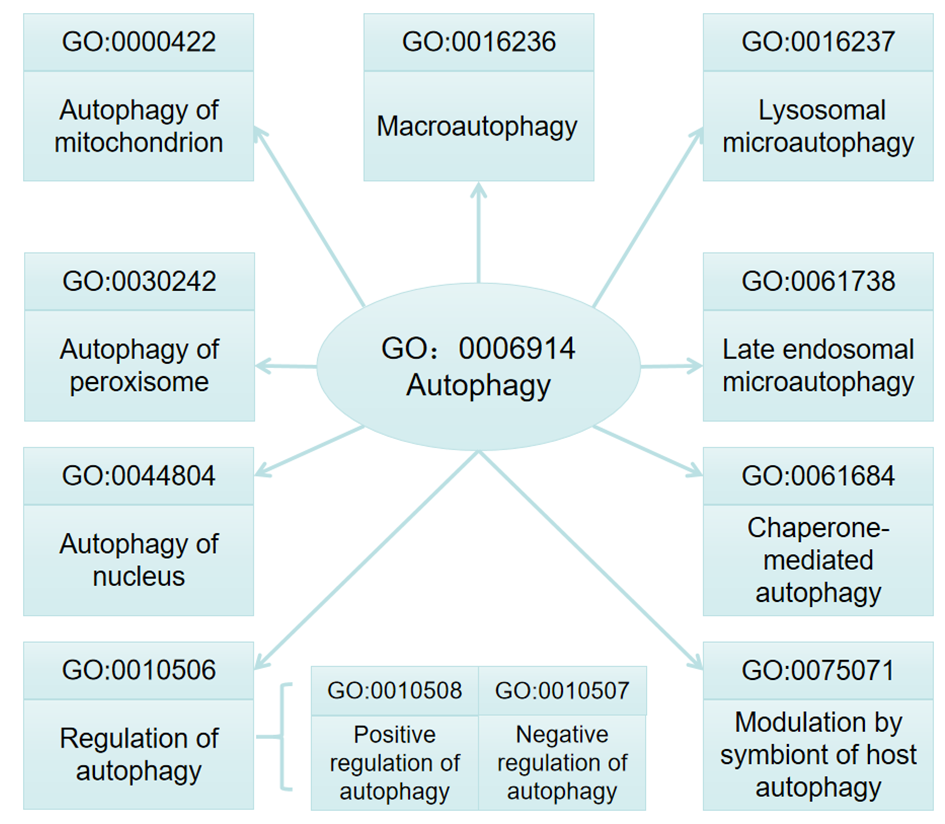

Supplement: Supplementary Figure 1 — Identification of autophagy-related gene sets (ARGSs). [file Image_1.TIF]

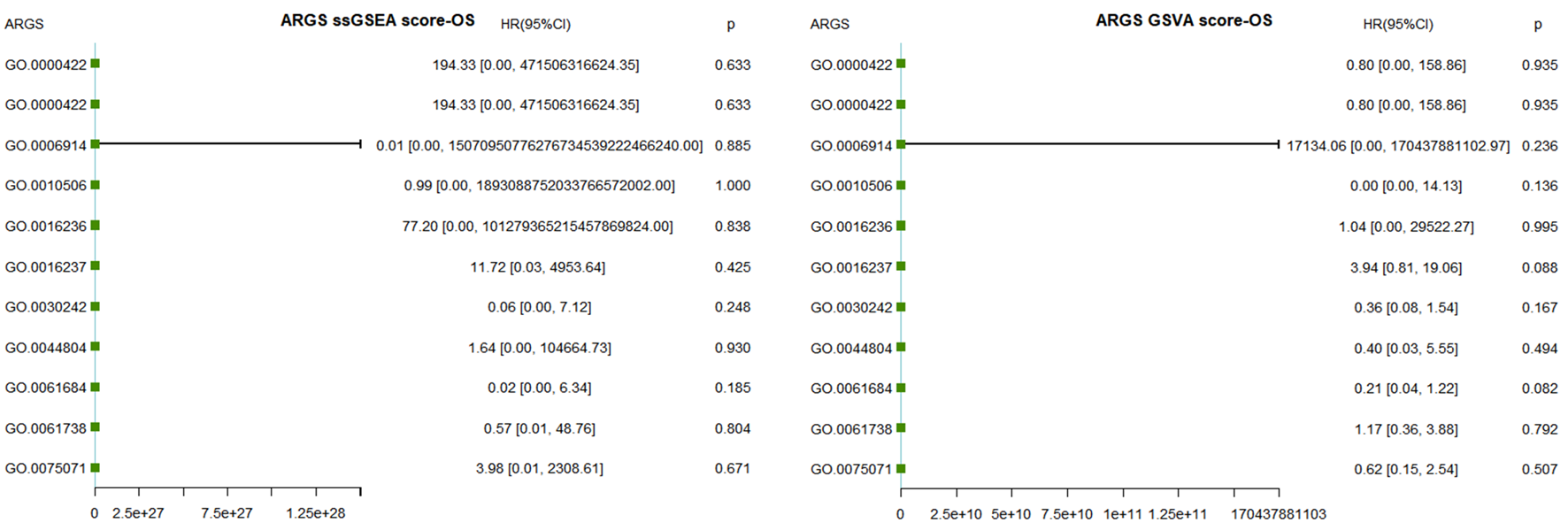

Supplement: Supplementary Figure 2 — Multivariate Cox regression analysis of ARGSs with overall survival. [file Image_2.TIF]

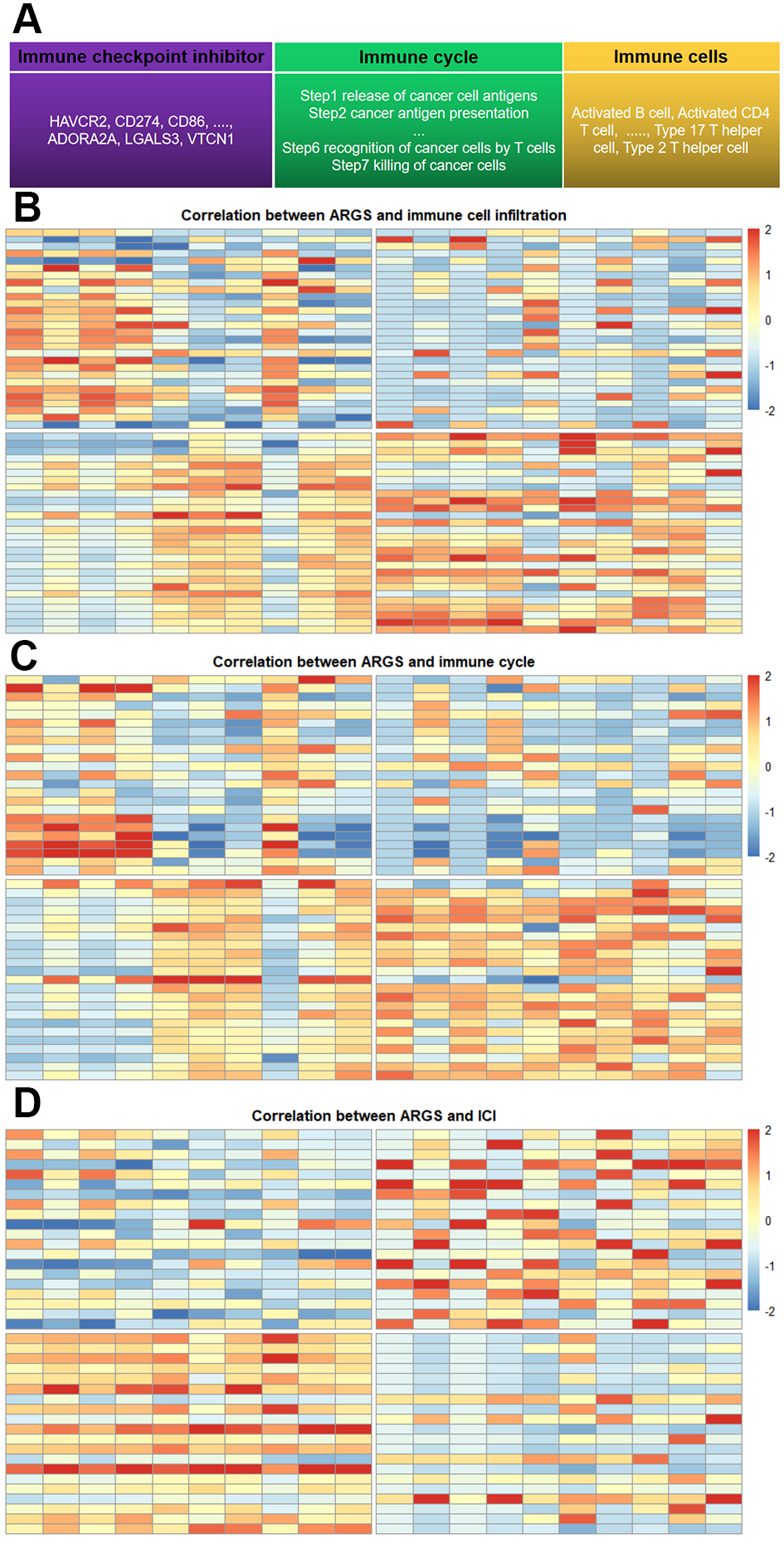

Supplement: Supplementary Figure 3 — Role of ARGS in predicting immune phenotypes. (A) The content of tumor immune microenvironment. (B) Correlations between ARGSs and infiltration levels of tumor-associated immune cells. (C) Correlations between ARGSs and immune cycle. (D) Correlations between ARGSs and immune checkpoint inhibitors. Upper left of (B–D) represent the r value while upper right represent p value by calculating ARGSs ssGSEA score; lower left represent r value while lower right represent p value by calculating ARGSs GSVA score. [file Image_3.TIF]

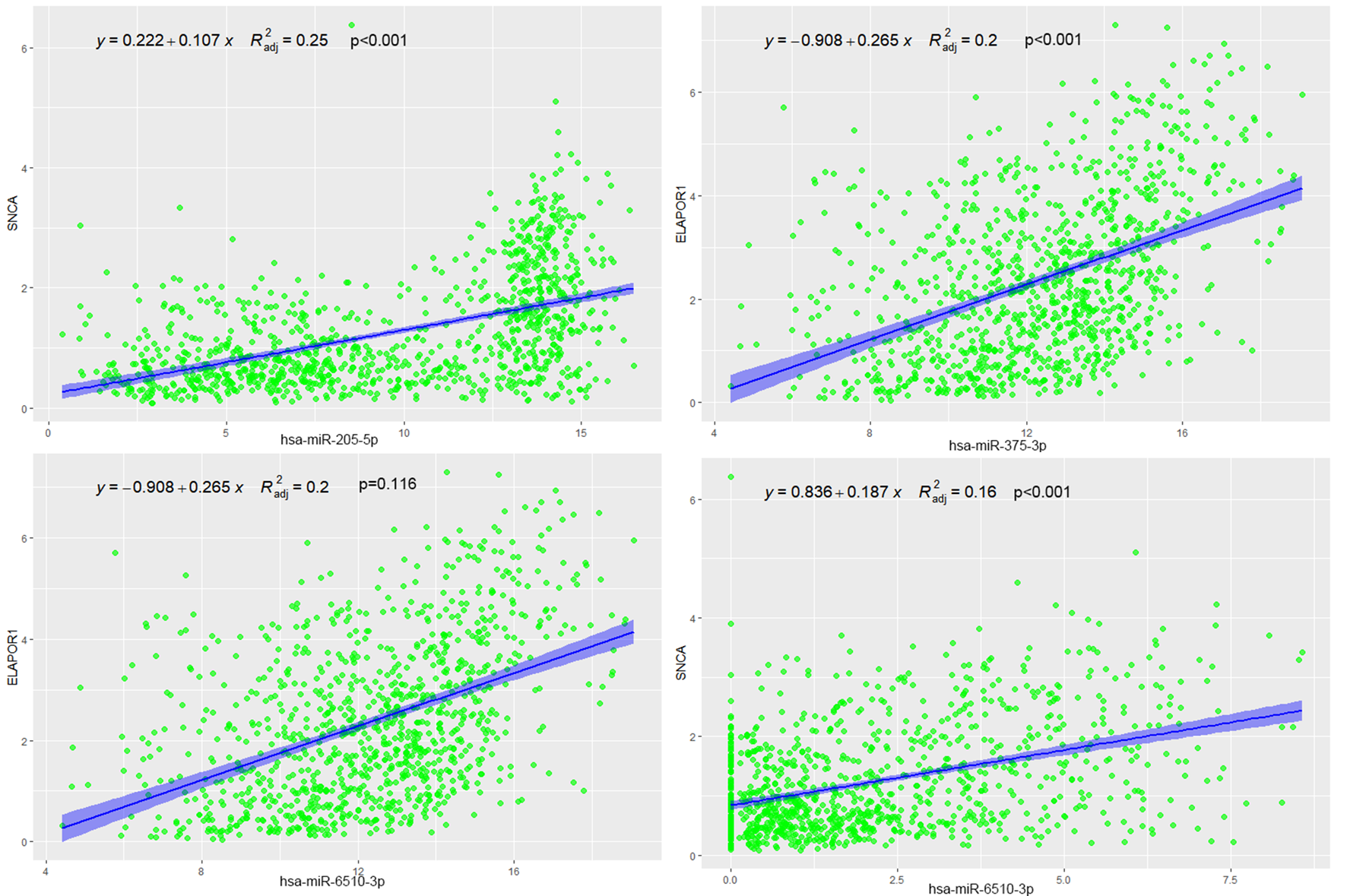

Supplement: Supplementary Figure 4 — Correlation analysis between other predicted miRNAs and ARmRNAs. [file Image_4.TIF]
